# The identification of a Distinct Astrocyte Subtype that Diminishes in Alzheimer’s Disease

**DOI:** 10.14336/AD.2024.0205-1

**Published:** 2024-03-04

**Authors:** Haichao Wei, Joseph Withrow, Jyotirmoy Rakshit, Faiz Ul Amin, Joshua Nahm, Francesca E. Mowry, Zhengmei Mao, Meenakshi B. Bhattacharjee, Jay-Jiguang Zhu, Yongjie Yang, Jia Qian Wu

**Affiliations:** ^1^The Department of Neurosurgery, McGovern Medical School, The University of Texas Health Science Center at Houston, Houston, TX, USA.; ^2^Center for Stem Cell and Regenerative Medicine, UT Brown Foundation Institute of Molecular Medicine, Houston, TX, USA.; ^3^Department of Neuroscience, Tufts University School of Medicine, Boston, MA, USA.; ^4^Department of Pathology and Laboratory Medicine, McGovern Medical School, The University of Texas Health Science Center at Houston, Houston, TX, USA.; ^5^MD Anderson Cancer Center UTHealth Graduate School of Biomedical Sciences, Houston, TX, USA.

**Keywords:** Alzheimer’s disease, astrocyte subpopulation, astrocyte heterogeneity, SnRNA-seq, amyloid beta, Tau

## Abstract

Alzheimer's disease (AD) is characterized by the presence of two hallmark pathologies: the accumulation of Amyloid beta (Aβ) and tau proteins in the brain. There is a growing body of evidence suggesting that astrocytes, a type of glial cell in the brain, play crucial roles in clearing Aβ and binding to tau proteins. However, due to the heterogeneity of astrocytes, the specific roles of different astrocyte subpopulations in response to Aβ and tau remain unclear. To enhance the understanding of astrocyte subpopulations in AD, we investigated astrocyte lineage cells based on single-nuclei transcriptomic data obtained from both human and mouse samples. We characterized the diversity of astrocytes and identified global and subpopulation-specific transcriptomic changes between control and AD samples. Our findings revealed the existence of a specific astrocyte subpopulation marked by low levels of *GFAP* and the presence of *AQP4* and *CD63* expression, which showed functional enrichment in Aβ clearance and tau protein binding, and diminished in AD. We verified this type of astrocytes in mouse models and in AD patient brain samples. Furthermore, our research also unveiled significant alterations of the ligand-receptor interactions between astrocytes and other cell types. These changes underscore the complex interplay between astrocytes and neighboring cells in the context of AD. Overall, our work gives insights into astrocyte heterogeneity in the context of AD and reveals a distinct astrocyte subpopulation that holds potential for therapeutic interventions in AD. Targeting specific astrocyte subpopulations may offer new avenues for the development of novel treatments for AD.

## INTRODUCTION

Astrocyte is a predominant type of glial cell in the central nervous system (CNS), representing a population of functionally diverse cells [1-8]. The functions of astrocytes include maintaining the blood-brain barrier, providing trophic, and metabolic factors to neurons, recycling neurotransmitters, regulating synaptogenesis and synaptic transmission, and maintaining homeostasis of neural networks [8]. During injuries and/or neurological diseases, astrocytes become reactive and proliferate, which may be protective or causative for pathologic phenotypes in a context-dependent manner. Astrocytes are involved in the regulation of many neurological diseases [9, 10].

In Alzheimer’s disease (AD), astrocytes play a complex role [11]. They respond to the accumulation of Amyloid beta (Aβ) and tau by releasing inflammatory molecules and other factors that can contribute to neuronal damage. However, astrocytes also have protective functions and can help clear amyloid beta and internalize tau from the brain [12-17]. Aβ and tau proteins are both considered as biomarkers of AD [18]. Aβ is a peptide that plays a central role in the development of AD. It is derived from amyloid precursor protein (APP). In AD, Aβ accumulates in the brain, forming neuritic plaques among neurons. Tau protein, on the other hand, is a microtubule-associated protein that stabilizes the structure of nerve cells. In AD and other tauopathies, tau protein undergoes abnormal modifications, forming tangled structures called neurofibrillary tangles. These tangles impair the transport of essential nutrients and other molecules within the cells, disrupting their normal functions. Astrocytes can express several tau-binding proteins on their cell surface, including heparan sulfate proteoglycans (HSPGs), which can bind to tau protein and promote its clearance from the brain [19].

Studies have revealed distinct subtypes of astrocytes based on their gene expression profiles, morphology, location, and functional properties. These subtypes can have different roles in supporting neuronal function, regulating synapse formation and plasticity, modulating blood flow, maintaining ion homeostasis, and influencing the immune response in the brain [20, 21]. In AD, reactive astrocytes are often found surrounding amyloid plaques, which are aggregates of amyloid beta protein. The increment of *GFAP* (Glial fibrillary acidic protein) expression in reactive astrocytes in AD is thought to be associated with astrocyte dysfunction and impaired ability to provide support to neurons [22]. It was reported that the reactive astrocytes may lose their normal supportive functions and instead contribute to neuroinflammation and the progression of neuronal damage in AD [23, 24]. Still little is known about the effect of the molecular and functional heterogeneous subtypes of astrocytes and their contributions in AD.

To investigate the heterogeneity of astrocytes and how they change during AD progression, we analyzed a number of publicly available large datasets of single-nuclei RNA sequencing (snRNA-seq) studies from humans and mouse AD and control samples and focused on astrocyte lineage cells. We identified a population marked by *CD63*, *AQP4* (Aquaporin 4) expression, and low level of *GFAP* expression that is present in normal brain but diminishes in AD samples in both human and mouse. Gene set enrichment analysis revealed that this population is enriched in functions related to Aβ clearance and tau protein binding. Importantly we subsequently verified this subpopulation with immunohistochemistry of specific marker genes in normal human and AD prefrontal cortex samples, as well as mouse AD models.

## MATERIALS AND METHODS

### Data Mining of snRNA-seq in the Mouse/human Alzheimer disease

Published data are downloaded from the Gene Expression Omnibus (GEO) and Synapse. Data from Habib, N. et al. (10X Genomics, GEO: GSE143758), Zhou et al. (10X Genomics, GEO: GSE140399)[25], Lau SF et al. (10X Genomics, GEO: GSE157827)[26], Morabito S et al. (10X Genomics, GEO: GSE174367)[27], and Grubman, A. et al. (10X Genomics, GSE138852.)[28] were downloaded in fastq format from GEO. Data from Mathys H. et al. (10X Genomics, syn18485175) were downloaded in fastq format from Synapse (www.synapse.org/#! Synapse:syn18485175)[29]. Raw snRNA-Seq reads of datasets were processed individually according to 10x Genomics CellRanger software (cellranger- 3.0.2) and mapped to the mm10/GRCm38 genome with default parameters.

### SnRNA-seq data processing, clustering, and visualization

Count matrices for each dataset were imported into Seurat R package[30]. Count matrices from dataset syn18485175 were integrated by cellranger aggr function, and the other studies were integrated by Seurat IntegrateData function. To reduce the dimensionality of the snRNA-Seq dataset, principal component analysis (PCA) was performed on an integrated data matrix and the top 30 PCs were selected for downstream analysis. Clustering was then performed by Seurat’s FindClusters function, with resolution res = 0.5 and then visualization was performed by 2D UMAP plots. Conventional markers described were used to categorize each cluster into known biological cell types: pan-neuron markers (*STMN2*, *SNAP25*, *ENO2*, *SYN1*, *SYT1*, *SNAP25*, *GRIN1*), Excitatory neurons (*SLC17A7*, *CAMK2A* and *NRGN*), Inhibitory neurons (*GAD1* and *GAD2*), Oligodendrocyte (*MBP*, *MOBP*, *PLP1* and *MOG*), OPC (*OLIG1*, *OLIG2*, *PDGFRA*, *SOX10*, *CSPG4*), Microglia (*CD68*, *AIF1*, *PTPRC* and *ITGAM*), Astrocyte (*AQP4*, *GFAP*, *ALDH1L1*, *SOX9*, *GJB6*, *SLC1A3*, *SLC1A2*, *ALDOC*, *S100B*, *NDRG2* and *ATP1B2*), and Endothelia (*FLT1*, *CLDN5*, and *ESAM*).

### The analysis of gene set enrichment and identification of subpopulation-specific marker in human dataset

The DEG (differentially expressed genes) for each subpopulation were analyzed by Seurat Findallmarker function. Gene sets considered in this analysis include all MSigDB C5 signature sets [31]. GSEA (gene set enrichment) was performed using the fgsea package (v1.16.0) with 1000 permutations [32]. The gene sets with P-value < 0.05 were considered significant. GSEA plots were produced using the 'plotEnrichment' function from the 'fgsea' R package.

To identify marker genes for the specific astrocyte subpopulation of interest, we employed two genes: one that distinguishes this astrocyte subpopulation from other cell types, and another gene that is uniquely expressed in the astrocyte subpopulation of interest. First, we compare cells with the GFAP low expression and diminished in AD with other cell types. Briefly, we extract the cells with the GFAP low expression and diminished in AD from astrocyte lineage cells, then rename them as “ast_cluster1”. DEGs between ast_cluater1 with other clusters were identified by Seurat Findmarker function. The intersection of DEGs across all clusters was acquired and presented in the Upset plots [33]. DEGs specific to the astrocyte subpopulation of interest were identified using the Seurat 'FindAllMarkers' function in astrocyte lineage cells. It's important to note that not all genes exhibiting high fold changes necessarily serve as marker genes. Therefore, we also assessed the distribution of DEGs across various clusters. We regarded genes that were uniquely expressed in a particular cluster as marker genes. We also observed that certain clusters have a low number of cells. Consequently, these clusters may not be suitable candidates for further investigation.

### Cell-cell communication analysis

Cell communication analysis was performed on control and Alzheimer datasets, using CellChat with default parameters and ligand-receptor interaction databases [34], separately. The interaction database was obtained from CellChatDB, which is integrated into the CellChat repository (https://github.com/sqjin/CellChat). We filtered out cell-cell communications when less than 10 cells per group were observed. The aggregated ligand-receptor interaction scores were calculated for each signaling pathway. We use mergeCellChat to integrate CellChat objects from control and Alzheimer samples. By utilizing pattern recognition techniques, we identified the prevailing incoming and outgoing signal patterns for each subpopulation in control and AD samples. We inferred the primary sources and targets of the signaling network within a specific pathway through network centrality analysis. The communication probability of a ligand-receptor pair between the control and AD samples is calculated using a law of mass action model that considers ligand and receptor concentrations, any known cofactor concentrations, and the number of cells in each cell type. Signaling pathways were considered active with p-value<0.05.

### Immunohistochemistry (IHC) of Human Brain Samples

Paraffin embedded tissue blocks of frontal lobe cortex were obtained from autopsy specimens of 3 patients with a pre-mortem clinical diagnosis of Alzheimer's Dementia. Control samples were obtained from 3 age-matched autopsy specimens without a diagnosis of Alzheimer’s Dementia.

Paraffin embedded frontal cortex sections 6 microns thick were deparaffinized, then heated in 0.5 mM citric acid at pH 6.0 for 20 minutes for antigen retrieval. All rinses were with Tris-buffered saline, pH 7.6 with 0.025% Triton X100. Sections were incubated with 10% normal donkey serum in phosphate buffered saline with 1% BSA (blocking solution) for 2 hours at room temperature. Sections were incubated with the primary antibodies [rat anti-GFAP (Invitrogen; cat #13-0300; 1:1000), and rabbit anti-CD63 (Thermofisher; cat # 25682-1AP; 1:1000), mouse anti-AQP4 (abcam; cat #Ab9512; 1:1000) diluted in blocking serum overnight at 4°C. Fluorescent conjugated secondary antibodies (TRITC donkey anti-rat, Jackson #712-025-150, 1:200 for GFAP; FITC Goat anti-rabbit, Invitrogen #A16024, 1:300 for CD63; CY5 Donkey anti-mouse, Jackson #715-175-151, 1:500 for AQP4) were applied for 120 minutes at room temperature. Sections were counterstained with DAPI (Santa Cruz Biotechnology; cat #SC3598; 1:1000) and cover slips were applied with Prolong Anti-Fade Gold reagent (Life Technologies). Three sets of slides were used for each experiment.

3×3 stitched images centered in frontal lobe subcortical white matter were captured using a Nikon AR Confocal Microscope with 20x objective with frame size at 2048 x 2048 and a bit depth of 12 (Nikon Instruments Inc., USA). For each sample, 1 image from 3 separate slides was used. The same acquisition settings were for all images. NIS elements general analysis 3 (Nikon NIS-Elements HC Ver 5.41.02 Build 1711, Nikon Instruments Inc., USA) was used to create a segmentation algorithm to quantify the number of cells with overlapping immunofluorescent signal of the 4 channels. The same segmentation parameters were applied to all images. To delineate GFAP-high and GFAP-low cells immunoreactivity (IR) was measured using an adaptation Zeppenfeld et al. method for AQP4 IR quantification [35]. The IR of GFAP binary objects was measured for all GFAP binary objects. The average IR value for all GFAP^+^ binary objects was calculated. DAPI^+^ GFAP^+^AQP4^+^ CD63^+^ cells were divided into GFAP-high and GFAP-low by the objects value relative to the average IR of all GFAP binary objects. Cells were considered DAPI^+^GFAP^high^ AQP4^+^CD63^+^ if the IR value for GFAP binary object was higher than the average IR value of all GFAP^+^ binary objects. Conversely, cells were considered DAPI^+^ GFAP^low^AQP4^+^CD63^+^ if the IR value for GFAP binary object was lower than the average IR value of all GFAP^+^ objects. The number of DAPI^+^GFAP^low^AQP4^+^CD63^+^ objects, as well as the number of DAPI^+^GFAP^+^ objects was counted for all images.

### Immunohistochemistry (IHC) of mouse APP knock-In mouse brain samples

The APP^NL-F/NL-F^ knock-in (APP^NL-F^) mice brain sections from Dr. Yongjie Yang lab at Tufts University were used in this study [36]. Both male and female mice were used in experiments and were assigned to experimental groups in age- and sex-matched manner. Aged C57BJ/6 wild-type control (WT) control mice were also obtained from the same institution. After washing with PBS-T (0.2% Triton X-100 in PBS; Sigma-Aldrich) three times, tissue sections on slides were blocked with PBS-T containing 10% donkey serum for 2 hours, and then incubated with primary antibodies diluted in blocking solution at 4^?^C overnight. Sections were incubated with the primary antibodies (rat anti-GFAP (Invitrogen; cat #13-0300; 1:300), mouse anti-AQP4 (abcam; cat #Ab9512; 1:100) diluted in blocking serum overnight at 4°C. Fluorescent conjugated secondary antibodies (Donkey anti-rat-488 (Invitrogen, cat # SA5-10026) 1:300 for GFAP; CY5 Donkey anti-mouse, Jackson ImmunoResearch, cat #715-175-151, 1:300 for AQP4) were applied for 60 minutes at room temperature. After washing with 1X PBS sections were incubated for another 90 minutes with Rat Anti mouse CD63-APC conjugated antibody (BioLegend, cat# 143906, 1:200). Sections were counterstained with Hoechst 33342 (Sigma cat# 14533) and cover slips were applied with ProLong Gold anti-fade reagent (Invitrogen; cat# P36930). Similarly in another set of experiments the Rat anti mouse -CD31 (BioLegend; cat# 102502,1:300) antibody was used along with previously described Anti-AQP4 and Anti-CD 63 antibody. Secondary antibody Anti Rat-647 conjugated (Invitrogen cat# A21247) was used. In the study in checking the engulfment of Aβ, Rabbit anti- Aβ antibody (Abcam; cat# ab201061, 1:300) was used along with previously described Anti-GFAP and Anti-CD63 antibody. Secondary antibody Anti Rabbit-647 conjugated (Jackson ImmunoResearch, cat #715-175-152, 1:300) was used. Three sets of slides were used for each experiment. A secondary antibody only slides and only tissue slides without antibody were used to rule out the background signal as well as autofluorescence. Images were taken in Nikon AXR confocal microscope under 40X water immersion objective.

### Immunocytochemistry (ICC) for Aβ internalization study using primary astrocyte culture

Mouse primary astrocytes were isolated from neonatal (P-7) mice brain as described previously (Wei et.al; 2021). Cells were seeded in Laminin (Gibco, cat# 23017015) coated 2 chambered slides. After 24 hours of seeding cells were treated with FAM-Aβ42 (AnaSpec Inc. cat# AS-23526-01) for 2 hours. Thereafter, cells were fixed with 4% Paraformaldehyde (PFA) followed by blocking with 1% BSA and 22.52 mg/mL glycine dissolved in PBST (PBS+ 0.1% Tween 20). After blocking for 30 mins and constitutive washing, cells were incubated with primary antibody against GFAP (rat anti-GFAP; Invitrogen; cat #13-0300; 1:300) and CD63 (Goat Anti cd63; cat# AA 120-175) at 4°C overnight. After overnight incubation cells were washed and incubated with secondary antibodies (Donkey Anti-Rat Cy5; Jackson Immuno Research, cat # 712-175-153 and Donkey Anti Goat TRITC; cat# ABIN 336452) followed by counter staining with Hoechst 33342 (Sigma cat# 14533) and mounting with ProLong Gold anti-fade reagent (Invitrogen; cat# P36930). A secondary antibody-only slide and cell- only slide without antibody were used to rule out the background signal as well as autofluorescence. Images were taken in Nikon AXR confocal microscope under 40X water immersion objective.

### Statistical Analyses

The data were performed by an independent t-test under a normal distribution. The data were presented as mean ± standard error of the mean (SEM), and statistical significance was determined for p-values less than 0.05 using the t-test. 'n' denotes the number of sections for cell counting, and the data underwent t-test analysis. Significance levels are indicated by asterisk(s): *** p < 0.001. Further statistical details can be found in the figure legends.

## RESULTS

### SnRNA-seq atlases of cell populations in Alzheimer’s disease

In this study, we analyzed publicly available snRNA-seq data of AD, including two mouse and four human AD datasets in total. Initially, we focused on one human and one mouse datasets. The snRNA-seq data of the mouse AD dataset are hippocampus and cortex from 21 control and 16 5xFAD with age 1.5-19.6 months (GSE143758) [25]. The human AD dataset is derived from the prefrontal cortex (syn18485175, 24 individuals with no AD pathology and 24 individuals with AD-pathology) [29]. We analyzed these data and defined the astrocyte lineage cells by known markers (Supplementary Fig. 1). The mouse dataset contained astrocytes, oligodendrocytes, and endothelial cells, but a low proportion of neurons and cells with both OPC (Oligodendrocyte precursor cells) and astrocyte markers (referred to as Cells with O/A markers) (Supplementary Fig. 1A). 6 clusters (clusters 5, 7, 9, 14, 15, and 16) in the mouse AD dataset expressed astrocyte genes (e.g., *Slc1a2*, *Slc1a3*, *Aldoc*, *S100b*, and *Ndrg2*). cluster11 expressed endothelial genes (e.g., *Cldn5*, *Flt1*, and *Esam*) and cluster8 expressed mature oligodendrocyte genes (e.g., *Plp1*, *Mbp*, and *Mobp*). The human dataset (syn18485175) contains astrocytes, oligodendrocytes, neurons, and cells with O/A markers, and a low proportion of endothelial cells (cluster20) (Supplementary Fig. 1B). In the human dataset, cluster 8 (cells with O/A markers) expressed both OPCs marker genes *PDGFRA* and *CSPG4*, and astrocyte markers (e.g., *SLC1A2*, *ALDOC*, and *S100β*). Cluster 0 expressed markers of mature oligodendrocyte including *PLP1*, *MBP*, and *MOBP*. Two clusters (cluster6 and 21) in the human dataset have only astrocyte gene expression. 5 neuron clusters (clusters 9, 10, 14, 16, and 17) expressed inhibitory neurons (In) markers (e.g., *GAD1* and *GAD2*) and 11 neuron clusters (cluster 1, 2, 3, 4, 5, 7, 11, 12, 13, 15, and 18) expressed excitatory neurons (Ex) markers (e.g., *SLC17A7*, *CAMK2A*, and *NRGN*).

**Figure 1.**
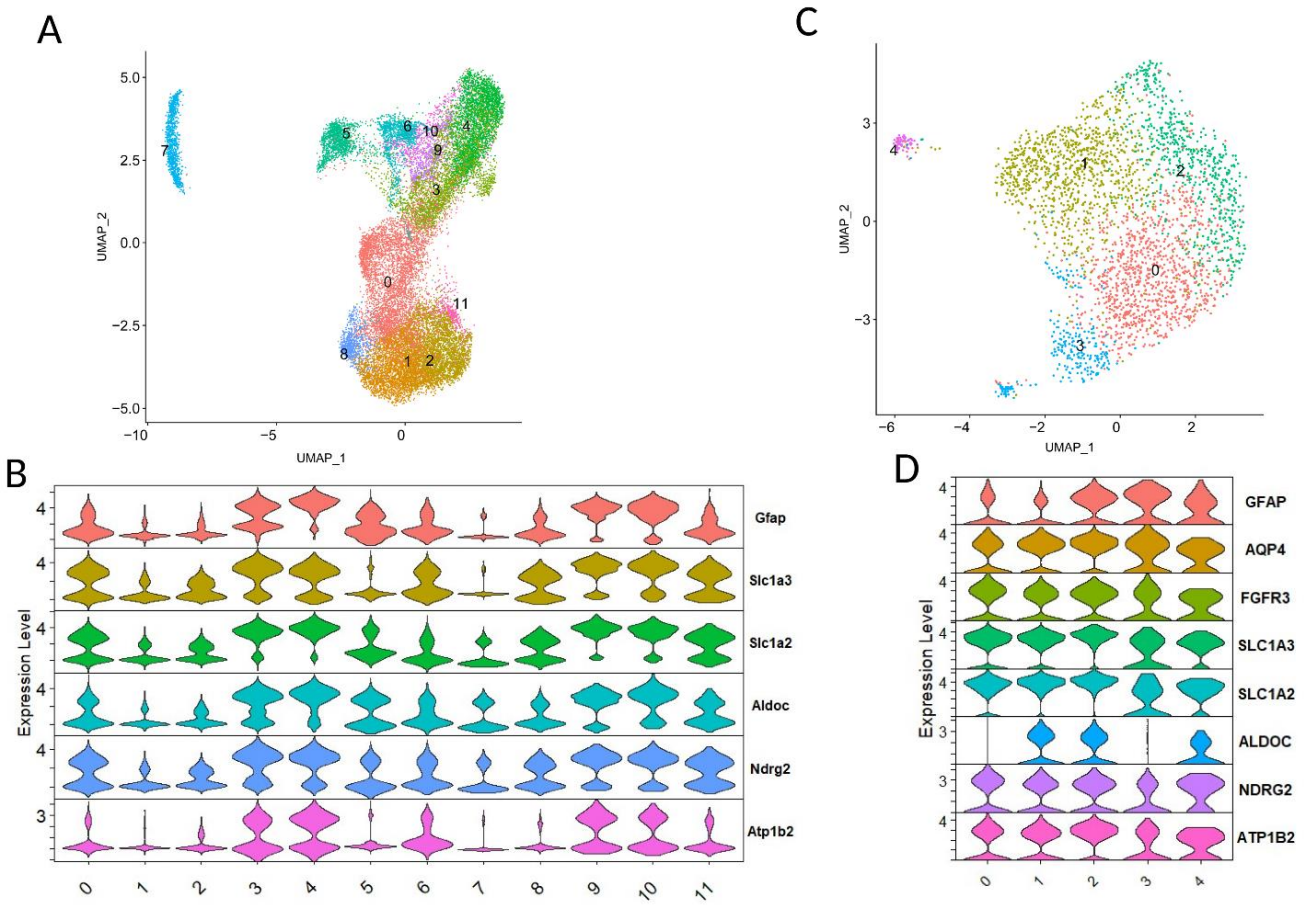
**Astrocyte lineage cells identified in published single nuclei RNA-seq datasets from GSE143758 (A and B) and syn18485175 (C and D)**. For each dataset, UMAP plots of astrocyte lineage cells and the expression of astrocyte specific marker. Dot size indicates the proportion of expressing cells, colored by standardized expression levels.

### The significantly decreased astrocyte subpopulation expressed GFAP at low level in both mouse and human AD datasets

**Figure 2.**
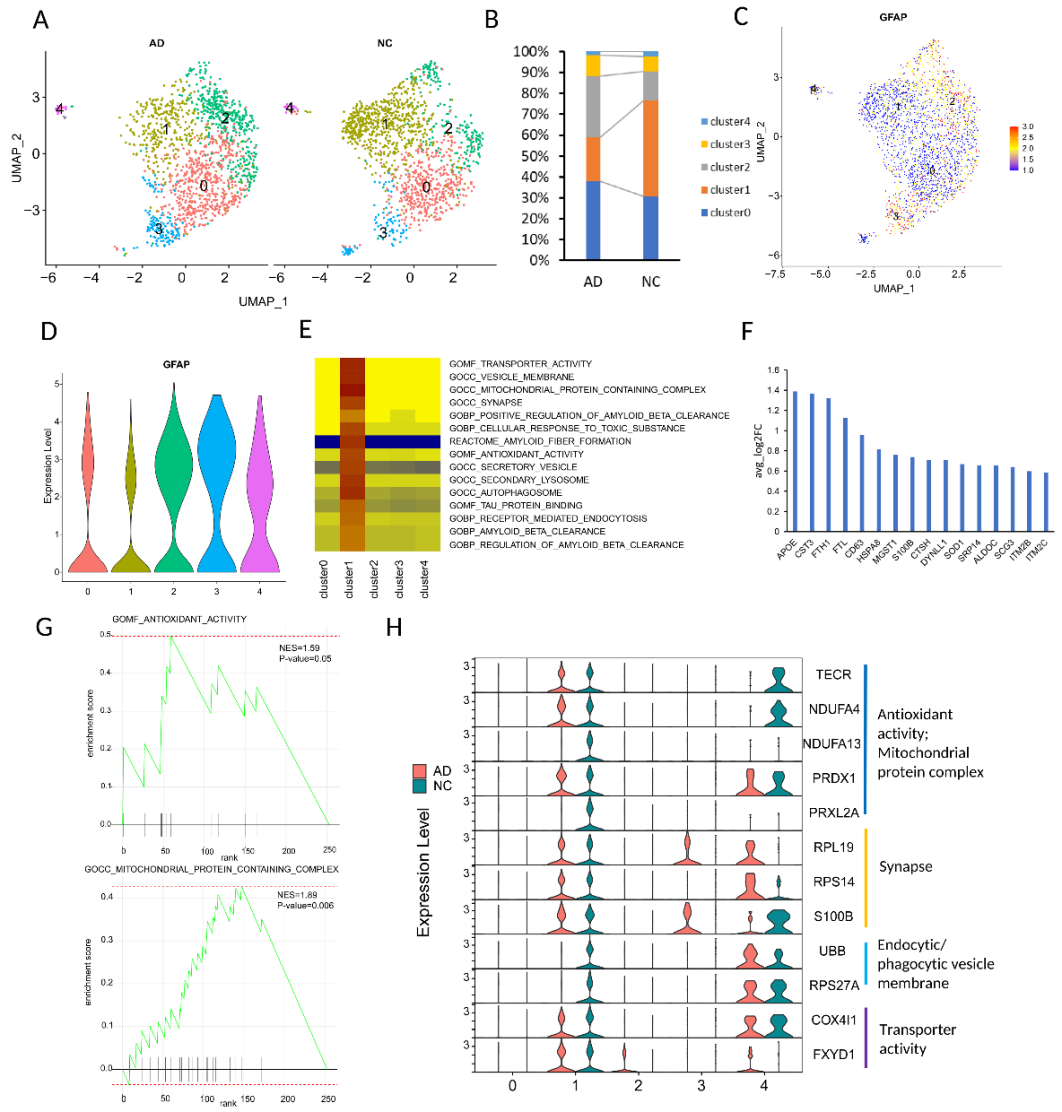
**The characteristics of astrocyte subpopulations from dataset syn18485175. (A)** The UMAP visualization of astrocyte lineage cells split into AD patients and controls. **(B)** The percentage of each Astrocyte subpopulations in control and AD samples. Colored according to cluster types. **(C)** UMAP plots of *GFAP* expression in astrocyte lineage cell populations. **(D)** Violin plot showing scale log-normalized read counts of *GFAP* expression. **(E)** GSEA results for astrocyte lineage cell clusters based on DEGs in each cluster. **(F)** The expression of secondary lysosome and secretory vesicle related genes. The foldchange was determined by comparing cells in the cluster 1 with those in all other cell populations. **(G)** Gene set of GSEA enriched in cluster1 compared with other astrocyte lineage cells. H, Expression levels of enriched genes in cluster1 related to antioxidant activity, mitochondrial protein complex, synapse, endocytic/phagocytic vesicle membrane, and transporter activity.

In order to characterize the heterogeneity of astrocytes in human and mouse AD samples, we clustered astrocyte lineage cells and identified the different astrocyte subpopulations. Figure 1 showed astrocytes were heterogeneous and had *GFAP*-low and *GFAP*-high expression patterns in both mouse and human datasets. To investigate the cell type diversity and changes in AD astrocyte cells, we analyzed cellular diversity with the Seurat clustering (Fig. 2 A-D and Supplementary Figure 2). Data from the human dataset contained five astrocyte subpopulations (cluster0-4), and the proportion of *GFAP*-positive cells in cluster0 and 2 increased whereas cells in cluster1 decreased (Fig. 2A and B). The Mouse AD dataset had 12 astrocyte subpopulations (clusters 0-11) and cells in cluster 7 were almost lost completely in AD datasets (Supplementary Fig. 2A and B). Supplementary 2C and D showed cells in cluster 7 had low expression of *Gfap*. We also found that cells in clusters 1 and 2 had low expression of *Gfap* but the proportion of cells in both clusters did not significantly change. All these results showed *GFAP* low astrocytes could be detected in control mice and humans. Additionally, there is a significant decrease in the proportion of a specific *GFAP*-low astrocyte subpopulation in AD samples, consistent with a previous study [25].

**Figure 3.**
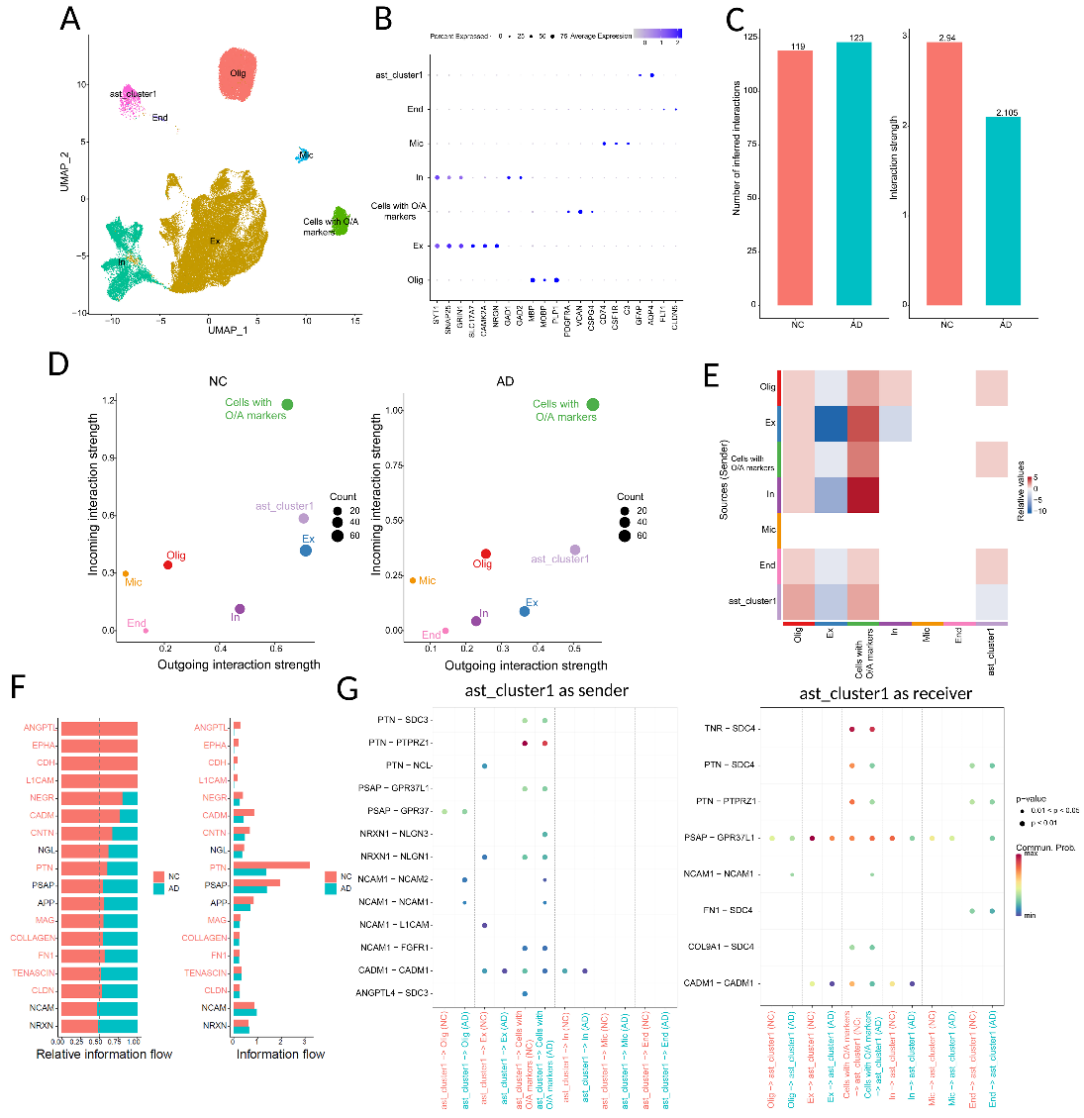
**Inferred intercellular communication network in AD samples from dataset syn18485175. (A)** UMAP visualization showing clustering of cells. Colors depict different clusters identified. In, inhibitory neuron; Ex, excitatory neuron; Oli, oligodendrocyte; End, endothelial; Mic, microglia. **(B)** Dot plot displaying the average expression levels of marker genes identified in each cluster. Dot size indicates the proportion of expressing cells, colored by standardized expression levels. **(C)** Total number and strength of interactions in control and AD groups. **(D)** Scatter plot of incoming and outgoing interaction strength of each cell population. **(E)** Heatmap of differential interaction strength in the control group compared to AD group. **(F)** Overall information flow of each signaling pathway in control and AD groups. The top signaling pathways, colored red, were more enriched in control samples. **(G)** Comparison of the significant ligand-receptor pairs between control and AD (ast_cluster1 as sender and receiver), dot color reflects communication probabilities and dot size represents p-values computed from one-sided permutation test.

### The astrocyte subpopulation of interest is implicated in Aβ clearance and Tau protein binding

To gain insight into the functional characteristics of the subpopulations of interest, we started the analysis by identifying the Differentially Expressed Genes (DEGs) through a comparative analysis of each subpopulation against all other astrocyte lineage cells. Subsequently, we performed a Gene Set Enrichment Analysis (GSEA) to elucidate the functional profiles specific to each subpopulation. Normal astrocytes play a crucial role in supporting synapses, which are the connections between neurons in the brain [37]. They contribute to various synaptic functions, including structural support, neurotransmitter uptake and recycling, clearance of metabolic waste, and maintenance of ion balance. In the human dataset, cells in cluster1 (ast_cluster1) had low expression of GFAP and significantly reduced in AD. GSEA revealed specific enrichments in "AMYLOID BETA CLEARANCE," "AUTOLYSOSOME," "SECONDARY LYSOSOME," "SECRETORY VESICLE," and "TAU PROTEIN BINDING" within ast_cluster1 (Fig. 2E). The degradation Aβ and tau protein-related genes enriched in ast_cluster1 (such as *APOE*, *CTSH*, *BRI2* (*ITM2b*), *HSPA8*, *HSPA9* and *HSPA1A*) (Fig. 2F) [38-40]. Further, we observed that cells in ast_cluster1 also exhibited functional enrichment associated with SYNAPSE, ANTIOXIDANT, TRANSPORTER, MITOCHONDRIAL and VESICLE MEMBERANE, and CELLULAR RESPONSE TO TOXIC SUBSANCE (examples in Fig. 2G and Supplementary Fig. 3). Aβ could be endocytosed by clathrin-coated vesicles in astrocytes [41] and endocytic/phagocytic vesicle membrane-related genes specifically expressed in ast_cluster1 (e.g., *UBB* and *RPS27A*) (Fig. 2H). Antioxidants are compounds that help counteract the damaging effects of oxidative stress by neutralizing harmful free radicals in the body. In the context of AD, antioxidants have been extensively studied for their potential neuroprotective effects and their ability to mitigate oxidative damage in the brain [42].

Mitochondrial dysfunction and oxidative stress have been implicated in the development and progression of AD. The mitochondria, responsible for generating energy within cells, can malfunction, leading to increased production of reactive oxygen species (ROS) and subsequent oxidative damage [43]. Notably, antioxidant activity and mitochondrial protein complex-related genes, such as *TECR*, *NDUFA4*, *NDUFA13*, *PRDX1*, and *PRXL2A*, were specifically enriched in ast_cluster1 and are involved in these pathways (Fig. 2H). Astrocytes reside in close proximity to synapses and hold a pivotal role in the regulation of synaptic function [44]. They are actively engaged in multiple essential facets of synaptic transmission, encompassing the control of neurotransmitter levels and the preservation of the extracellular environment surrounding synapses. Additionally, it is noteworthy that certain genes associated with synapse functionality, such as *RPL19*, *RPS14*, and *S100B*, as well as genes linked to transporter activity, such as *COX4L1*, and *FXYD1*, have been identified as enriched in ast_cluster1 (Fig. 2H). These findings highlight the potential importance of ast_cluster1 and their involvement in amyloid beta clearance, tau protein regulation, antioxidant activity, mitochondrial function, and synaptic support in the context of AD.

### Cell communications between the astrocyte subpopulation of interest and other cell types in AD

In order to obtain the communication of the astrocyte subpopulation of interest with other cell types in the human dataset, we compared ast_cluster1 with other cell types (Fig. 3 A and B). Cellchat utilizes a curated database of known ligand-receptor interactions to predict potential communication networks among cell populations [34]. Cellchat analysis revealed 119 total ligand-receptor interactions in the control group and 123 interactions in the AD group (Fig. 3C). The total interaction strength of the AD group was lower than that of the control group (Fig. 3C). Comparing the incoming and outgoing pathways, ast_cluster1 was the major signal sender and cells with O/A markers were the major signal receivers in both control and AD dataset (Fig. 3D). To study how the cell-cell communication changes between AD and control, we computed the differential number of interactions for both outgoing and incoming signaling of pairwise cell groups between AD and control (Fig. 3E). Compared to the AD sample, we observed that the signals sent from ast_cluster1 to oligodendrocyte or cells with both astrocyte and OPC markers increased and signals from ast_cluster1 to excitatory neuron decreased (Fig. 3E). To identify the common and differential signaling pathways, we compared the overall information flow for each signaling pathway. Figure 3F shows that although many signaling pathways were found in both control and AD groups, four signaling pathways (ANGPTL (Angiopoietin-like protein), EPHA (EPH receptor A), CDH (congenital diaphragmatic hernia), L1CAM (L1 Cell Adhesion Molecule)) were specifically active in control, suggesting these pathways might play important role in maintaining normal brain functions. Cellchat identified ligand-receptor pair ANGPTL4-SDC3 as the most significant signaling specific in control, contributing to the communication from ast_cluster1 to cells with both astrocyte and OPC markers (Fig. 3G). Ligand-receptor pairs NCAM1-L1CAM, NRXN1-NLGN1 and PTN-NCL were found to act as major signaling from ast_cluster1 to excitatory neurons specific in control (Fig. 3G). Furthermore, we also observed significant changes in signaling pathways when ast_cluster1 acted as the receiver. For example, we identified a decrease in interactions such as PTN-SDC4, PTN-PRPRZ1, and FN1-SDC4 between the communication of endothelial cells and ast_cluster1, as well as between cells with O/A markers and ast_cluster1 in AD samples compared to the control group (Fig. 3G).

**Figure 4.**
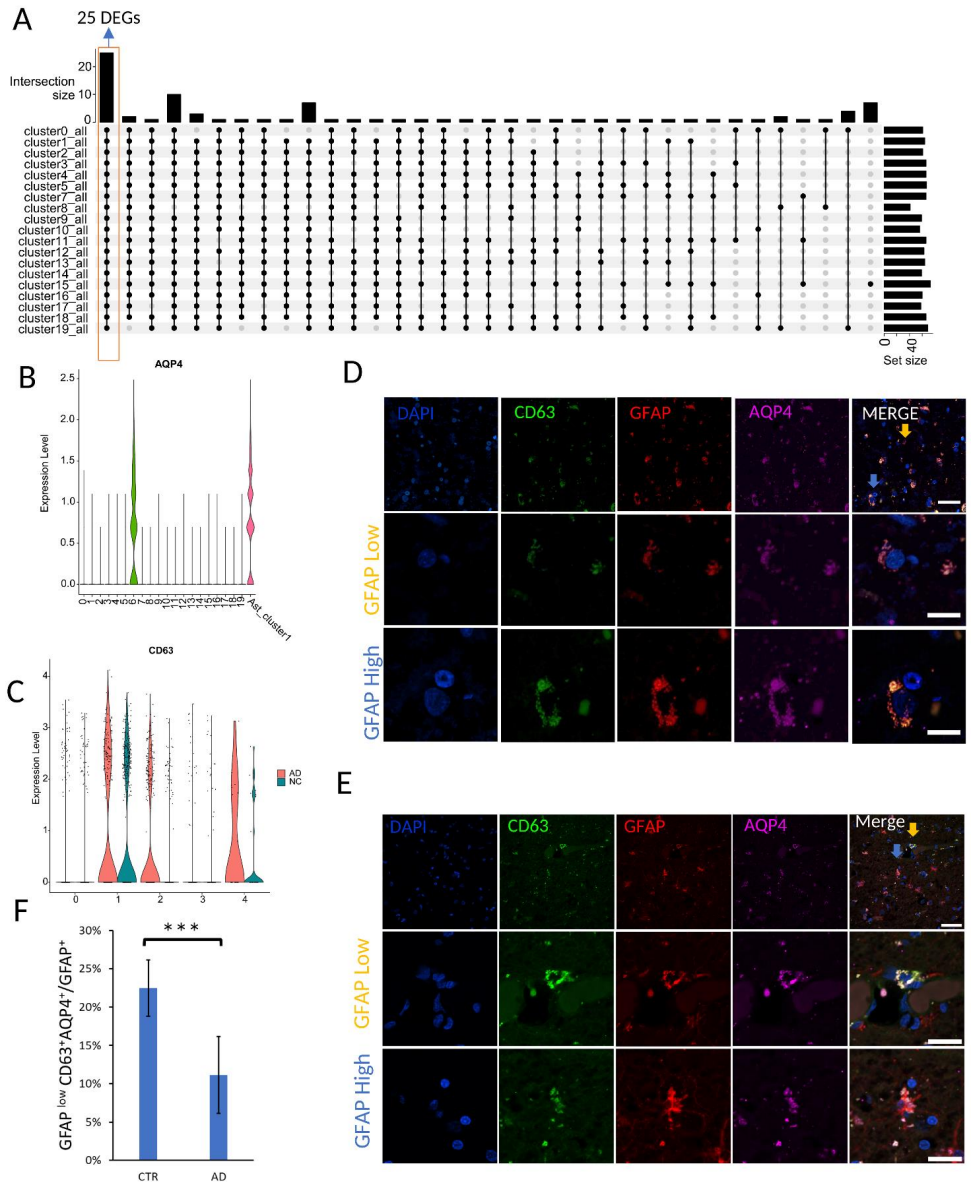
**The identification of *GFAP*^low^*AQP4*^+^*CD63*^+^ astrocyte subpopulation. (A)** UpSet plots showing the overlap of DEGs compared astrocyte cluster1 with all other cell types. 25 DEGs were shared by all groups. The vertical bar plot displays the intersection size, the dot plot illustrates the set participation in the intersection, and the horizontal bar plot represents the set sizes. **(B)** Vlnplots of *AQP4* expression in dataset syn18485175. The y axis shows the log-scale normalized read counts. **(C)** Vlnplots of *CD63* expression in AD and control samples. The y axis shows the log-scale normalized reads count. **(D-E)** Example of astrocytes that co-expressed GFAP (red), AQP4(pink), and CD63 (green) in human control (D) and AD samples (E). Cell nuclei are shown in blue (DAPI). The first row in a panel provides an overview of the tissue. Yellow arrow indicates GFAP^low^AQP4^+^CD63^+^ astrocyte and blue arrow indicates GFAP^high^AQP4^+^CD63^+^ astrocyte (Scale bar 40 µm). The second row shows a magnified view of GFAP^low^AQP4^+^CD63^+^ astrocytes (labeled by yellow arrows) and the third row show a magnified view of GFAP^high^AQP4^+^CD63^+^ astrocyte (labeled by blue arrows) (Scale bar 20 µm). F, The percentage of GFAP^low^AQP4^+^CD63^+^ cells in human control (n=7) and AD (n=9) sections. p-Value is determined by t-test, Data are presented as mean ± SEM. *** p< 0.001.

### Identification of marker genes in astrocyte subpopulation of interest

To identify the marker genes for the above-mentioned *GFAP*-low astrocyte subpopulation of interest, we compared cells in ast_cluster1 with each of other cell type. We found 25 up-regulated genes (*ADGRV1*, *AGT*, *AHCYL1*, *AQP4*, *ATP1A2*, *CLU*, *COL5A3*, *CPE*, *CST3*, *F3*, *FGFR3*, *GJA1*, *GJB6*, *GLUL*, *HEPN1*, *MT1E*, *MT1G*, *MT2A*, *NTRK2*, *PDGFRB*, *PLPP3*, *SLC14A1*, *SLC1A2*, *SLC1A3*, and *TUBB2B*) in clsuter1 that are common in all comparisons (Fig. 4A). Among these, *AQP4* is significantly upregulated in astrocyte lineage cells and not detected in other cell types (Fig. 4B). *AQP4* is involved in various astrocytic functions [9]. Iliff JJ et al. found that the deletion of *AQP4* suppressed the clearance of Aβ [45]. Next, we compared ast_cluster1 with all other astrocyte lineage cell clusters from the human dataset. We found the expression of *CD63* was specifically enriched in cluster 1 (ast_cluster1) (Fig. 4C). While some genes, such as *APOE* and *CST3*, exhibited high fold changes in cluster 1 compared to other clusters, they are also present in multiple other clusters, thus not suitable markers. CD63 is a tetraspanin protein and known to predominantly label intracellular endosomal intraluminal vesicles and exosomes that are secreted into the extracellular space [46]. CD63 interacts with a range of proteins, including integrins and the Src family tyrosine kinases Lyn and Hck. These interactions play a pivotal role in influencing intracellular signal transduction pathways crucial for processes such as vesicle trafficking and exosome release [47]. Jaiswal et al. demonstrated its specific localization to the lysosomal membrane [48]. Previous studies also showed astrocytes could release CD63-positive exosome and bind extracellular Aβ proteins to help Aβ clearance [49-52]. Although cluster4 had *CD63* expression, cluster4 contained few cells (Fig. 2A). Astrocyte lineage cell cluster2 also had *CD63* expression, but *CD63* only could be detected in AD samples and GFAP is expressed at a high level in this cluster. We also investigated the proportion of GFAP^low^AQP4^+^CD63^+^ cells in both control and AD samples. In the control astrocyte lineage cells, we found that majority of the GFAP^low^AQP4^+^CD63^+^ cells were in cluster 1. Together, these results showed *CD63* and *AQP4* can be used as markers for astrocyte subpopulations of interest with low *GFAP* expression.

### Validation of GFAP^low^AQP4^+^CD63^+^ subpopulation in human brain

To verify and further characterize the GFAP^low^AQP4^+^CD63^+^ subpopulation *in vivo*, we performed immunohistochemistry (IHC) using antibodies against GFAP, CD63, and AQP4 in control and AD samples (Fig. 4D and E). Based on the relation of the GFAP immunoreactivity (IR) value of the GFAP^+^AQP4^+^ CD63^+^ cells to the average IR value of all GFAP-positive cells (Methods), we separated the GFAP^+^AQP4^+^CD63^+^ cells into two subgroups: GFAP^low^AQP4^+^CD63^+^ and GFAP^high^AQP4^+^CD63^+^ cells. Our immunostaining results confirmed the presence of GFAP^low^AQP4^+^CD63^+^ subpopulations in both AD patients and control subjects (Fig. 4D-F). We further quantified the proportion of all GFAP^+^ cells that belong to the GFAP^low^AQP4^+^CD63^+^ subpopulation. Interestingly, the proportion of GFAP^low^AQP4^+^CD63^+^ cells was significantly reduced in the AD group compared to the control group (Fig. 4F). These findings were consistent with the proportions observed in the snRNA-seq data from both the AD and Control groups, validating our findings that the GFAP^low^AQP4^+^CD63^+^ subpopulation is decreased *in vivo*. These results provide important information of astrocyte heterogeneity and compositions in AD human brain compared to control.

### The verification of the GFAP^low^AQP4^+^CD63^+^ cell population in mice

**Figure 5.**
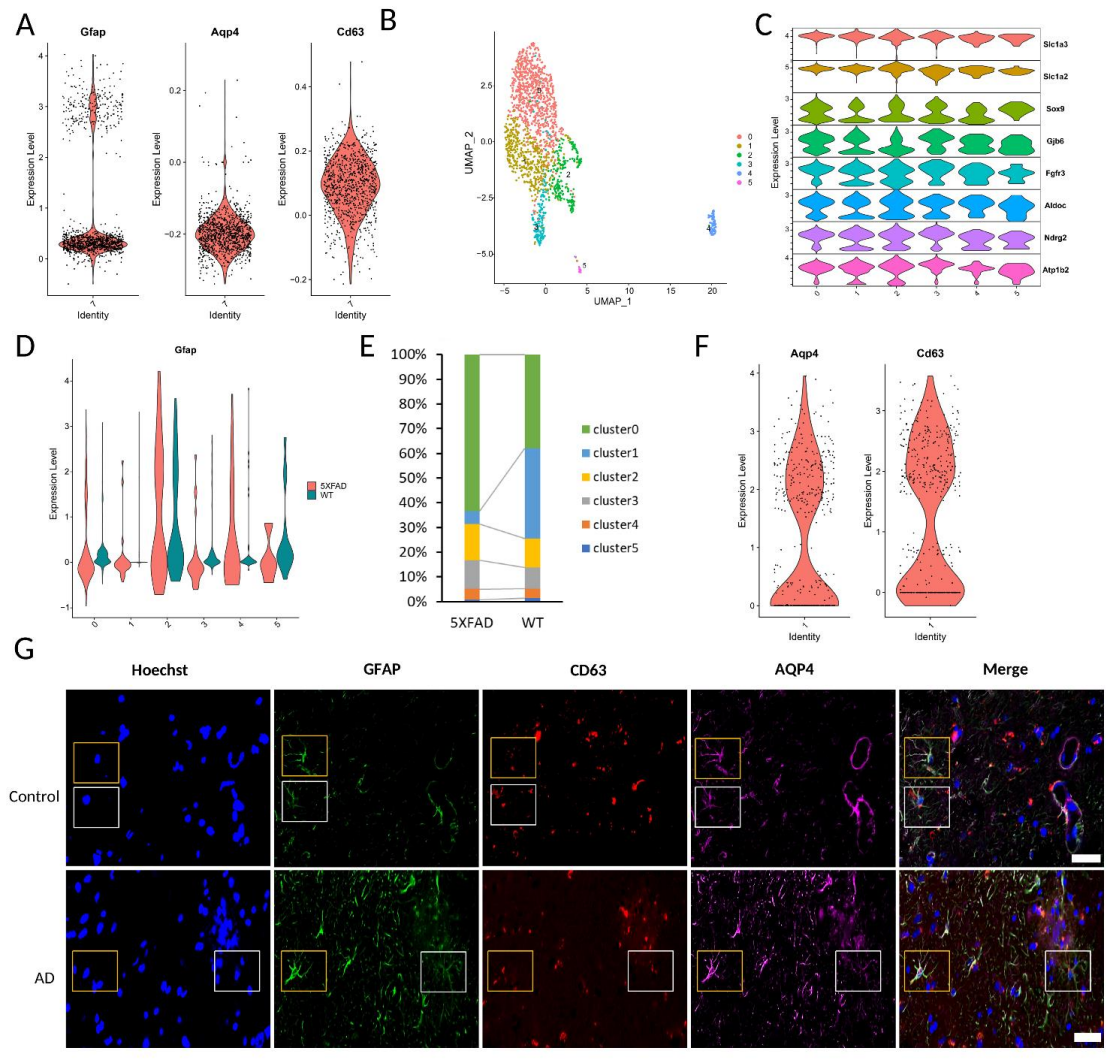
**The verification of *Gfap*^low^*Aqp4*^+^*Cd63*^+^ cells in mouse AD samples. (A)** Vlnplots of *Gfap*, *Aqp4*, and *Cd63* expression in cluster7 from dataset GSE143758. The y axis shows the log-scale normalized reads count. **(B)** UMAP plots of astrocyte lineage cells from Zhou et al. **(C)** Dot plot for markers characterizing the five selected astrocyte lineage cells. Dot size indicates the proportion of expressing cells, colored by standardized expression levels. **(D)** Vlnplots of *Gfap* expression in 5XFAD and control samples. The y axis shows the log-scale normalized reads count. **(E)** The percentage of subpopulations in 5XFAD and control samples. Colored according to cluster types. **(F)** Vlnplots of *Aqp4* and *Cd63* expression in 5XFAD and control samples. The y axis shows the log-scale normalized reads count. **(G)** Immunohistochemistry showing the presence of GFAP^high^AQP4^+^CD63^+^ (Yellow box) and GFAP^low^AQP4^+^CD63^+^ (White box) in the 27-month-old control (Upper panel) and 20-month AD (Lower panel) mouse prefrontal cortex (Scale bar 100 µm).

We further investigated if GFAP^low^AQP4^+^CD63^+^ cells are present within mouse brain samples. In the earlier part of the paper, we focused on the cells within cluster 7 of the mouse dataset from GSE143758. We observed that cells exhibiting low *Gfap* expression within cluster 7 demonstrated a significant reduction in AD samples. Notably, *Aqp4* and *Cd63* markers are also expressed within cluster 7 (Fig. 5A).

We extended our investigation to another mouse AD dataset [53] to validate these cell types. In this study, we only utilized the wild-type control and 5XFAD samples from GSE140511. We identified six distinct astrocyte lineage clusters (Fig. 5B and C). Notably, cells within cluster 1 exhibited low *Gfap* expression and significantly diminished in AD samples (Fig. 5D and E), paralleling our earlier findings. Additionally, cluster 1 is also marked by *Aqp4* and *Cd63* expression (Fig. 5F). Substantiating our findings, our immunohistochemical staining revealed the presence of GFAP^high^AQP4^+^CD63^+^ and GFAP^low^AQP4^+^ CD63^+^ cells within the cortex of late-onset AD model APP^NL-F/NL-F^ knock-in (APP NL-F) mice and in control sample [36] (Fig. 5G). This evidence collectively verified the existence of these cell types in both humans and mice.

**Figure 6.**
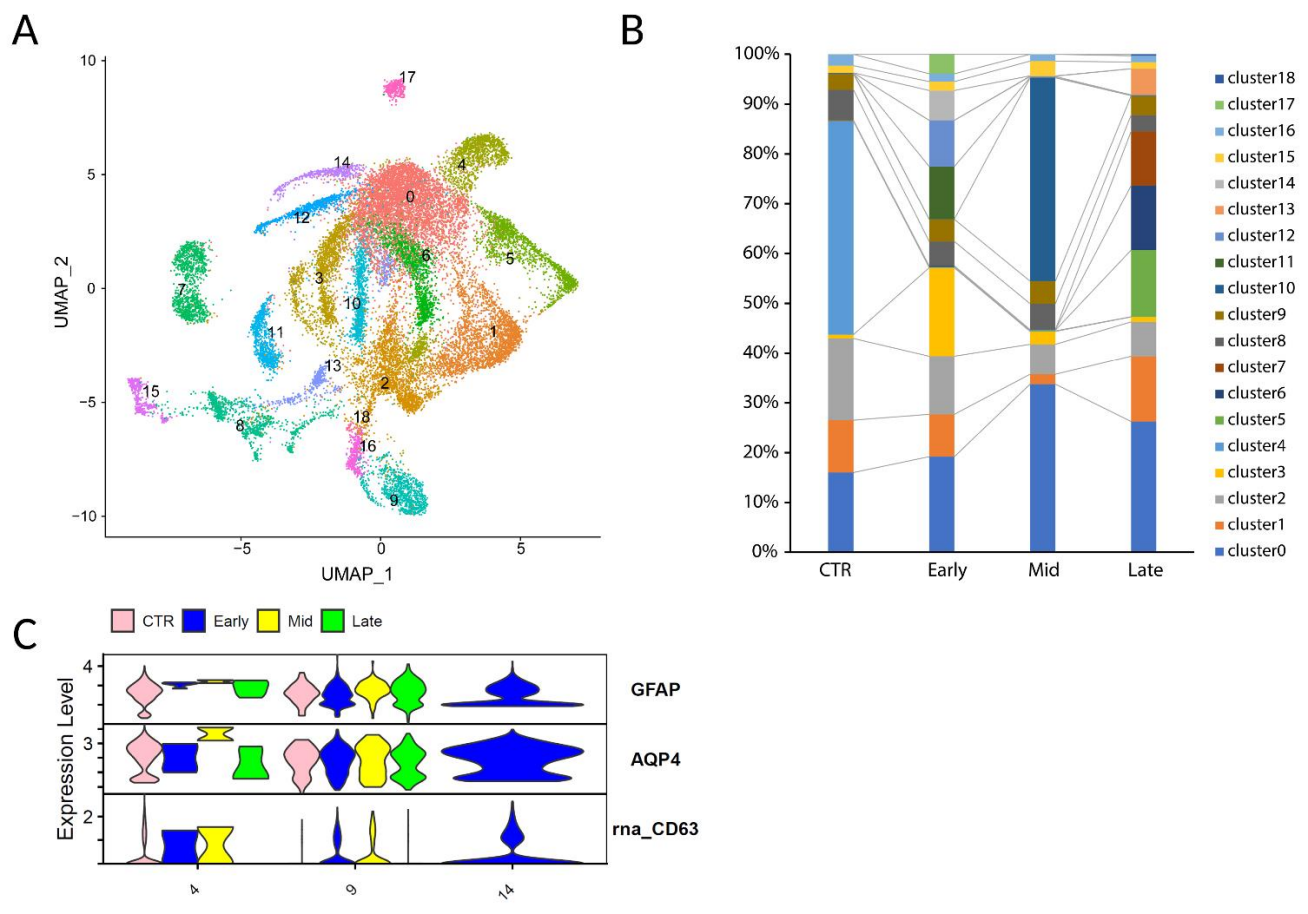
**The expression of GFAP^low^AQP4^+^CD63^+^ astrocyte in different stages of AD from Dataset GSE157827_and_GSE174367. (A)** UMAP plots of astrocyte lineage cells **(B)**, Proportion of astrocyte clusters in different stages of AD (CTR, Early, Mid, and Late) **(C)**. The expression of GFAP, AQP4, and CD63 in cluster4/9/14 separated by stages.

### GFAP^low^AQP4^+^CD63^+^ cell population further decreases with AD progression in human

The progression stages of AD are currently characterized by the presence and level of amyloid beta or tau accumulations in the brain. A question of significant interest is whether the population of GFAP^low^AQP4^+^ CD63^+^ cells decrease in the late stages of AD. To investigate this, we conducted an additional analysis utilizing snRNA-seq datasets from two independent studies from GSE157827 and GSE174367 where samples were obtained from the prefrontal cortex [26, 27]. These datasets were from subjects at different disease stages, including control subjects as well as those in the early, mid, and late stages of AD, as categorized by Braak stages. Our findings reveal that GFAP^low^AQP4^+^CD63^+^ cells were detectable in both healthy individuals and those in the early stages of AD. However, they notably diminished in the later stages of the disease (Fig. 6). These results strongly suggest that GFAP^low^AQP4^+^CD63^+^ cells may play a role in preserving brain function, and their decline in later AD stages could be a contributing factor to the progression of cognitive decline and neurodegeneration.

## DISCUSSION

Astrocytes, the predominant glial cells in CNS, exhibit diverse morphologies and functions [54]. Astrocyte subpopulations refer to distinct groups of astrocytes with unique gene expressions and functional enrichment profiles. There was evidence showing that specific subpopulations of astrocytes may exhibit altered characteristics in AD [55, 56]. Our previous research, as well as other studies, have demonstrated that relying on a single marker is insufficient for the accurate identification of cell types or subtypes [57, 58]. And notably the expression level of specific markers is also important for defining cell properties. Thereby, utilizing a panel of molecular markers and quantitative characterization of cell marker levels is the most effective approach.

In the present report, we analyzed publicly available large human snRNA-seq datasets and identified the astrocyte lineage cells by multiple known markers (Fig. 1C and D, and Supplementary Fig. 1B). We identified low *GFAP* expressing astrocyte populations and one of these populations is enriched in amyloid beta clearance and tau protein binding functions. And these cells diminish in the course of Alzheimer’s disease. Mouse dataset from GSE143758 also revealed that *Gfap*-low astrocytes decreased in AD and could be the potential source for both *Gfap*-high and DAA (disease-associated astrocytes), consistent with our findings [25]. However, the potential functions of these *Gfap*-low astrocytes were unclear and the set of markers defining this cell type was not investigated. We identified and verified the specific markers for this type of cell and verified in human and mouse brain samples. Moreover, cell-cell communication analysis results showed a significant difference in ligand-receptor interactions between control and AD samples.

AD is characterized by the accumulation of Aβ and tau neurofibrillary tangles in the brain. These pathologies disrupt normal brain function and contribute to the progressive cognitive decline and neurodegeneration seen in Alzheimer's disease. Aβ accumulation may trigger tau and subsequent neuronal damage [59]. Although the amyloid beta hypothesis has been a subject of debate in the field of AD research, there have been recent developments regarding a drug targeting Aβ that has shown some promise in slowing cognitive decline[60]. Previous studies have shown astrocytes have a crucial role in the metabolism of tau and the clearance of Aβ via the glymphatic system [15, 61-63]. Astrocytes in the brain and spinal cord comprise multiple subpopulations that display unique gene expression profiles, morphological characteristics, and functional properties. However, the specific roles of different astrocyte subpopulations in Aβ clearance and tau protein regulation are not yet fully understood.

*GFAP* is a commonly used marker for astrocytes, but its expression can vary among different subpopulations and under various conditions. In healthy people *GFAP* expression is usually relatively low [64]. *GFAP* expression undergoes significant upregulation in the presence of disease. Elevated *GFAP* expression and alterations in morphology represent the most frequently utilized markers, although should not be the only marker, for identifying reactive astrocytes [1]. These reactive astrocytes can be observed in a range of brain pathologies. Notably, astrocyte reactivity is primarily detected in brain regions strongly associated with synaptic loss and age-related cognitive decline, such as the hippocampus and frontal cortex [22]. In AD, astrocytes become activated in response to the presence of abnormal protein aggregates like beta-amyloid plaques. The astrocyte reactivity is primarily detected in brain regions strongly associated with synaptic loss and age-related cognitive decline, such as the hippocampus and frontal cortex [22]. This activation leads to alterations in gene expression and an increase in the production of various proteins. Elevated *GFAP* levels are commonly observed in the brains of individuals with AD. The insolubility of GFAP in the late stage of AD could potentially have harmful effects on astrocyte biology. It may overwhelm the protein degradation systems, including autophagy, and limit astrocytes’ ability to migrate and extend their processes [22, 65]. Therefore, targeting astrogliosis reduction could be a promising therapeutic approach in AD.

We propose that AD pathologies could be a result of loss of normal functions of astrocytes, as the particular subpopulation of astrocytes that decreases in AD may have an essential role in maintaining normal brain function. By examining the expression levels of *GFAP* in different astrocyte subtypes, we identified subpopulations with relatively low *GFAP* expression and verified that they existed in both mice and humans and diminished in AD. In syn18485175, we observed two clusters (clusters 0 and 1) with low *GFAP* expression. However, only the number of cells in cluster1 was significantly decreased in AD. Gene set enrichment showed cluster1 is enriched in amyloid beta clearance and tau protein binding, compared with all other astrocyte clusters. Also, amyloid-beta clearance and tau protein binding-related genes were enriched in cluster1. These findings suggest specific subpopulations of astrocytes play distinct roles in AD and cells in cluster1 may be involved in the regulation of amyloid beta and tau protein.

To study this subpopulation of astrocytes, we conducted comparisons with non-astrocyte cell types and other astrocyte lineage cells. Through this comparative analysis, we found that *AQP4* and *CD63* expressed in this subpopulation of interest (GFAP^low^AQP4^+^CD63^+^). AQP4 serves as the primary aquaporin in the mammalian brain, predominantly expresses in astrocytic endfeet, and acts as a key facilitator of water transport. Blood vessels play a crucial role in supplying neutrients and gathering metabolic waste in various organs, and their involvement has been implicated in several pathways related to Aβ clearance [66]. Within astrocyte end-feet, AQP4 functions as the principal water channel, orchestrating bidirectional fluid flux around blood vessels [67]. Prior research has indicated a reduction in astroglial AQP4 localization to perivascular endfoot processes in AD [35, 68]. The diminished perivascular AQP4 localization correlates with an escalation in local Aβ and tau pathological burden, alongside cognitive and functional decline in AD. Our finding is consistent with a reduction in GFAP^low^AQP4^+^CD63^+^ cells in the perivascular region (Supplementary Fig. 5). CD63 is a tetraspanin protein known to predominantly label intracellular endosomal intraluminal vesicles and exosomes that are secreted into the extracellular space. In a study by Men Y et al., CD63 reporter mouse model was generated to study the role of exosome in astrocyte-neuron interactions [69]. Deng Z et al. isolated astrocyte-derived CD63^+^ exosomes and demonstrated that these exosomes have the potential to reduce the toxicity of Aβ *in vitro* and clear Aβ plaques in AD mice [70]. Furthermore, astrocyte-derived exosomes are known to encapsulate neuroprotective proteins, including synapsin 1, as well as angiogenesis-associated molecules such as VEGF (vascular endothelial growth factor) [71]. These findings suggest that GFAP^low^AQP4^+^CD63^+^ cells could potentially serve as a source of CD63^+^ exosomes and play crucial roles in the context of maintaining brain health.

Astrocytes can help clear and phagocytose Aβ through their cell surface receptors, which recognize and bind to Aβ. Among a range of physiological functions, evidence suggests that astrocytes actively participate in phagocytic processes in the healthy brain [72]. Moreover, prior studies have demonstrated the ability of astrocytes to phagocytose Aβ [73]. Reactive astrocytes have been shown to establish a physical barrier that restricts the spread of amyloid beta; however, they may not efficiently in clearing Aβ [22]. We observed Aβ plaque has been internalized and colocalized with the GFAP^low^/CD63^+^ cells more so than the GFAP^high^/CD63^+^ cells *in vivo* and in astrocyte culture (Supplementary Fig. 6). Additionally, Sanchez-Mico et al. found impaired astrocyte phagocytosis in peri-plaque astrocytes [74]. The potential underlying reasons for the significant decrease in these GFAP^low^AQP4^+^CD63^+^ astrocytes in AD include the possibility that the GFAP^low^ astrocytes near the plaques may senesce within the AD brain. Alternatively, these cells could have undergone transformation into other cell types, such as high-*GFAP*-expressing astrocytes.

In addition to snRNA-seq data from syn18485175, we also observed such astrocyte subpopulation (GFAP^low^AQP4^+^CD63) significantly decreased in mouse AD samples from GSE143758, dataset. Importantly, we also verified this subpopulation in both human and mouse prefrontal cortex samples by immunostaining. Quantification using confocal microscope in human samples confirmed a significant reduction in the abundance of these cells in AD samples compared to control samples. The co-expression of *AQP4* and *CD63* in *GFAP* low-expressing cells may indicate their involvement in processes related to water transport and intracellular vesicle trafficking, which could be relevant to amyloid beta clearance mechanisms. These results highlight the significance of investigating specific astrocyte subpopulations in AD and their potential contributions to amyloid beta clearance.

Cell-to-cell communication plays a crucial role in the central nervous system. In the context of AD, alterations in cell-cell communication between astrocytes and other cells can have significant implications for disease progression. We performed cellchat analysis to investigate the difference in the communication of ast_cluster1 with other cell types between control and AD. We found cells in ast_cluster1 were a major source for signaling senders, and four signaling pathways (ANGPTL, EPHA, CDH, and L1CAM) were specific in control. Ast_cluster1 produces molecules factors that modulate Aβ deposition and clearance, including heparan sulfate proteoglycans and syndecan3 (SDC3). HSPGs and SDC3 can bind to Aβ and promote its clearance from the brain. NCAM1 and L1CAM have been implicated in cell adhesion, neurite outgrowth, and synaptic plasticity, which are processes that are disrupted in Alzheimer's disease. A previous study showed *L1cam* was expressed in the hippocampus of wild-type but not AD mice [75]. After transducing *L1cam* in the hippocampus and occipital cortex of AD mice, amyloid-β plaques were reduced. These findings suggest that the interactions between neurons and astrocytes, facilitated by NCAM1 and L1CAM, could potentially influence the clearance of amyloid-beta plaques in the brain. In addition, cells within ast_cluster1 also function as receivers in cell-cell communication processes. In comparison to control samples, there is a decrease in interactions such as PTN-SDC4 and FN1-SDC4 between endothelial cells and ast_cluster1, as well as between cells with O/A markers and ast_cluster1 in AD samples. Notably, *SDC4* is predominantly expressed in astrocytes and is involved in exosome biogenesis [76, 77]. These findings suggest that the regulation of exosome biogenesis in cells within ast_cluster1 may be influenced by the signaling communication between endothelial cells with astrocytes, and between cells with O/A markers with astrocytes.

In this study, AD samples were prepared using single nuclei isolation, which theoretically allows for the examination of all cell types present in the tissue. However, when comparing the proportions of different cell types across both human studies, we observed variations. We analyzed another human AD snRNA-seq dataset from the entorhinal cortex (GSE138852, six AD patients and six controls) [28]. We observed that cells in syn18485175 contained a greater diversity of excitatory neurons (11 Clusters) and inhibitory neurons (5 clusters) compared to the findings in GSE138852 which has three IN clusters (cluster 5, 6, and 8) and two Ex clusters (cluster 3 and 4) (supplementary Fig. 4A and B) [28]. Notably, all Ex clusters in the GSE138852 dataset showed a reduction in AD, whereas in the syn18485175 dataset, two Ex clusters (cluster 1 and 5) increased in AD, and five Ex clusters (clusters 4, 7, 11, 13, and 15) decreased in AD. Regarding astrocyte lineage cell analysis, we observed the presence of GFAP^low^AQP4^+^CD63^+^ cells from GSE138852 dataset (supplementary Fig. 4C and D). However, this subpopulation was not significantly decreased in the GSE138852 dataset. The discrepancies may be attributed to the low astrocyte numbers in the GSE138852 and brain regional difference. The methods of sample preparation may also play a role. In order to understand the dynamics of GFAP^low^AQP4^+^CD63^+^ cells in different stages of AD, we combined another two human AD snRNA-seq dataset diagnosed by braak stages from prefrontal cortex. Our findings showed GFAP^low^AQP4^+^CD63^+^ cells could be detected in control or early staged AD but lower in late stage of AD. Overall, our work gives significant insights to an astrocyte subpopulation that diminishes in Alzheimer’s disease. Better understanding of the astrocyte heterogeneity and distinct functions of the subpopulations may hold promise for therapeutic interventions for Alzheimer's disease in the future.

## Supplementary Materials

The Supplementary data can be found online at: www.aginganddisease.org/EN/10.14336/AD.2024.0205-1.

## Data Availability

The data that support the findings of this study are available in GEO at www.ncbi.nlm.nih.gov/geo/ and www.synapse.org/#!Synapse reference number GSE 143758, GSE140399, GSE157827, GSE174367, GSE138852, and syn18485175.
