# Supplementary material for: The identification of a Distinct Astrocyte Subtype that Diminishes in Alzheimer’s Disease
Source: Aging Dis. 2024 Mar 4;15(6):2752–69. doi: 10.14336/AD.2024.0205-1 (PMC11567244; doi:10.14336/AD.2024.0205-1)
Supplement: Supplementary file 1 — The Supplementary data can be found online at: www.aginganddisease.org/EN/10.14336/AD.2024.0205-1. [file AD-15-6-2752-s.pdf]

SUPPLEMENTARY DATA

The identification of a Distinct Astrocyte Subtype that Diminishes in Alzheimer's Disease

Haichao Wei, Joseph Withrow, Jyotirmoy Rakshit, Faiz Ul Amin, Joshua Nahm, Francesca E. Mowry, Zhengmei Mao, Meenakshi B. Bhattacharjee, Jay-Jiguang Zhu, Yongjie Yang, Jia Qian Wu

© 2024. Wei H et al. Published online at http://www.aginganddisease.org/EN/10.14336/AD.2024.0205-1

SUPPLEMENTARY DATA

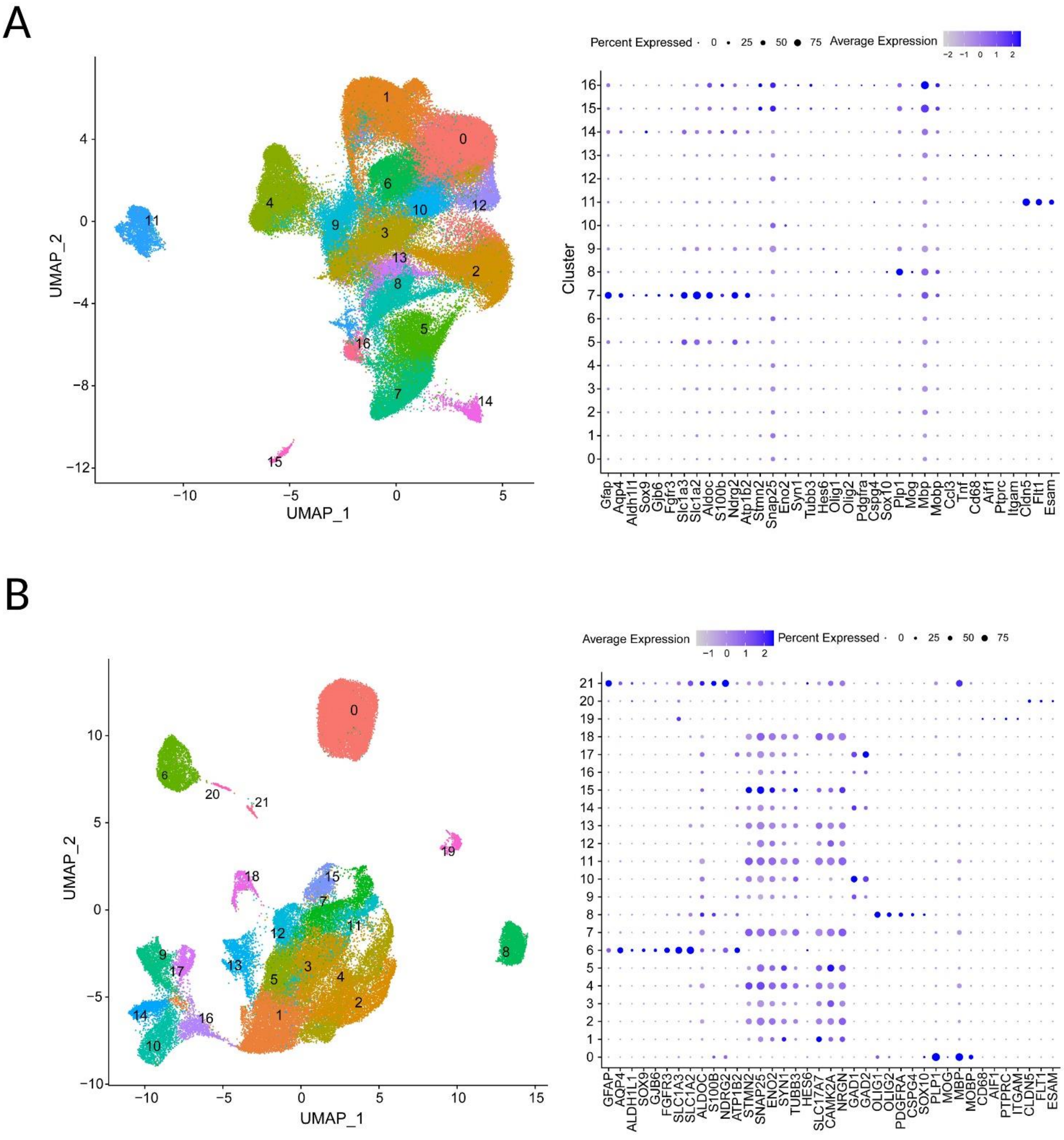

Supplementary Figure 1. cell type clusters identified from analyzing single nuclei RNA-seq datasets from GSE143758 (A) and syn18485175 (B). For each dataset, UMAP plots of all nuclei and the expression of various cell markers. Dot size indicates the proportion of expressing cells, colored by standardized expression levels.

© 2024. Wei H et al. Published online at http://www.aginganddisease.org/EN/10.14336/AD.2024.0205-1

SUPPLEMENTARY DATA

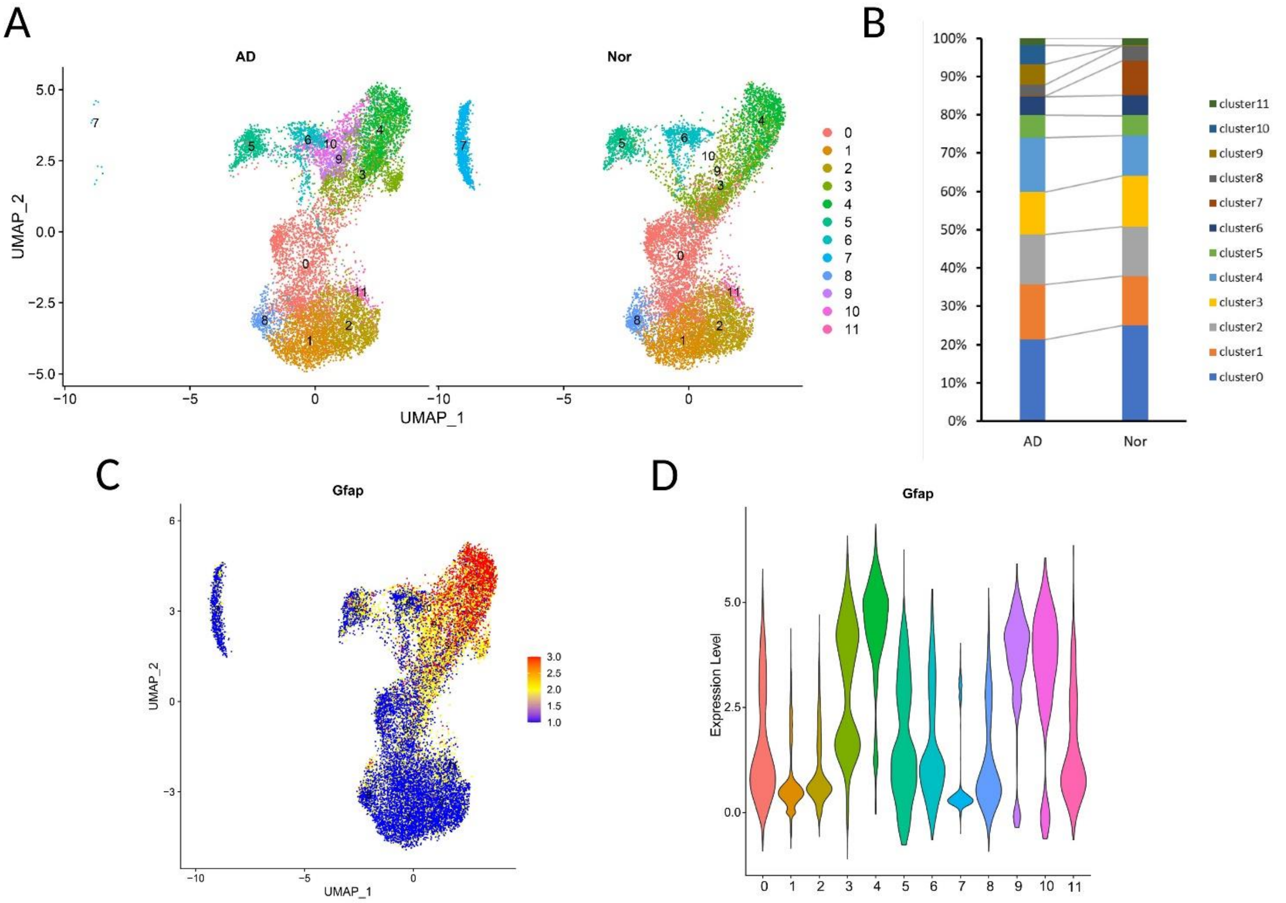

Supplementary Figure 2. Astrocyte subpopulations identified from analyzing dataset GSE143758. (A) The UMAP visualization of astrocyte lineage cells split into AD mice and controls. (B) The percentage of each Astrocyte subpopulations in control and AD samples. Colored according to cluster types. (C) UMAP plots of *GFAP* expression in astrocyte lineage cell populations. D, Violin plot showing scale log-normalized read counts of *GFAP* expression.

© 2024. Wei H et al. Published online at http://www.aginganddisease.org/EN/10.14336/AD.2024.0205-1

SUPPLEMENTARY DATA

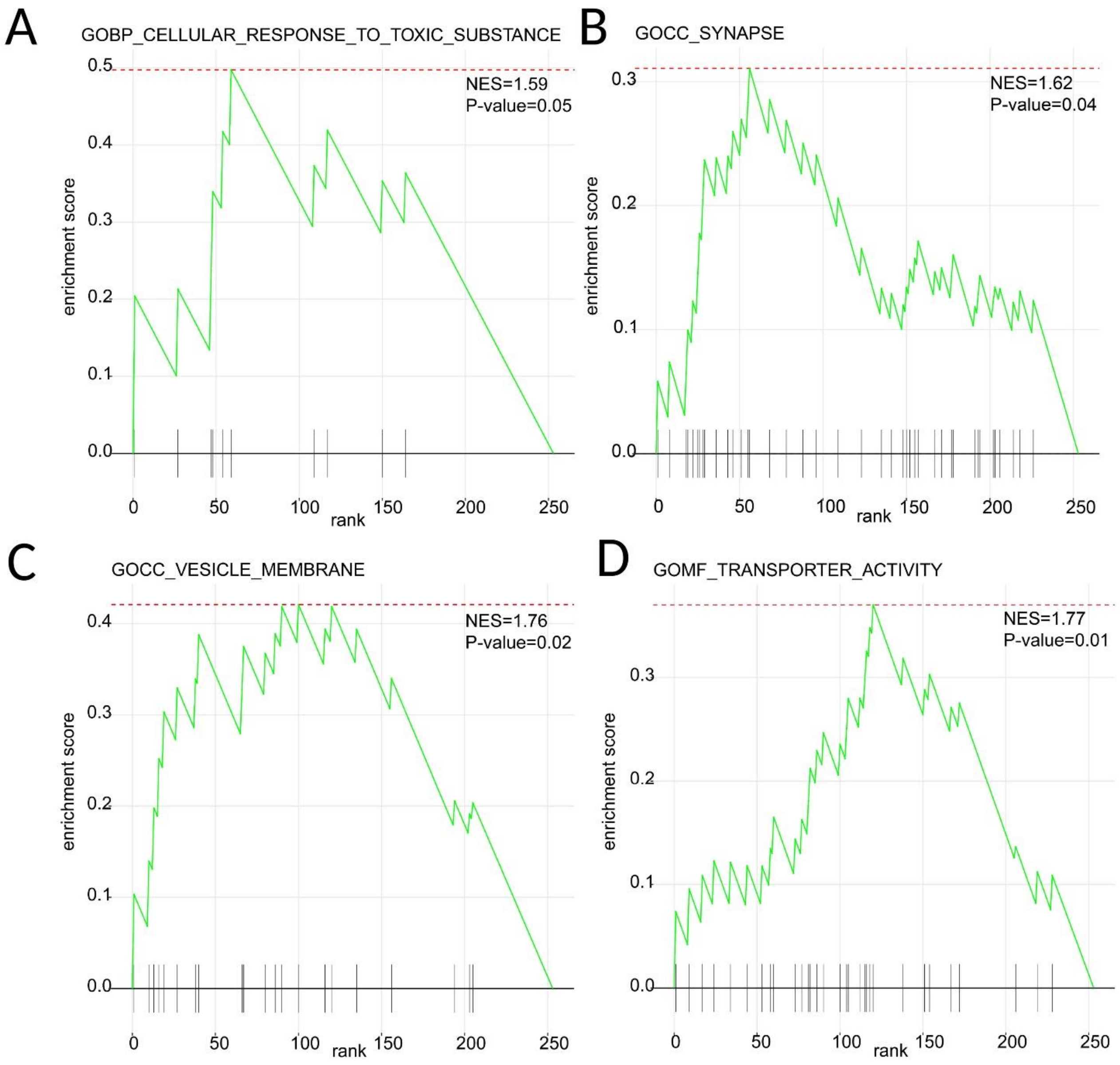

Supplementary Figure 3. The functional enrichment in ast_cluster1 cells from dataset syn18485175.

© 2024. Wei H et al. Published online at http://www.aginganddisease.org/EN/10.14336/AD.2024.0205-1

SUPPLEMENTARY DATA

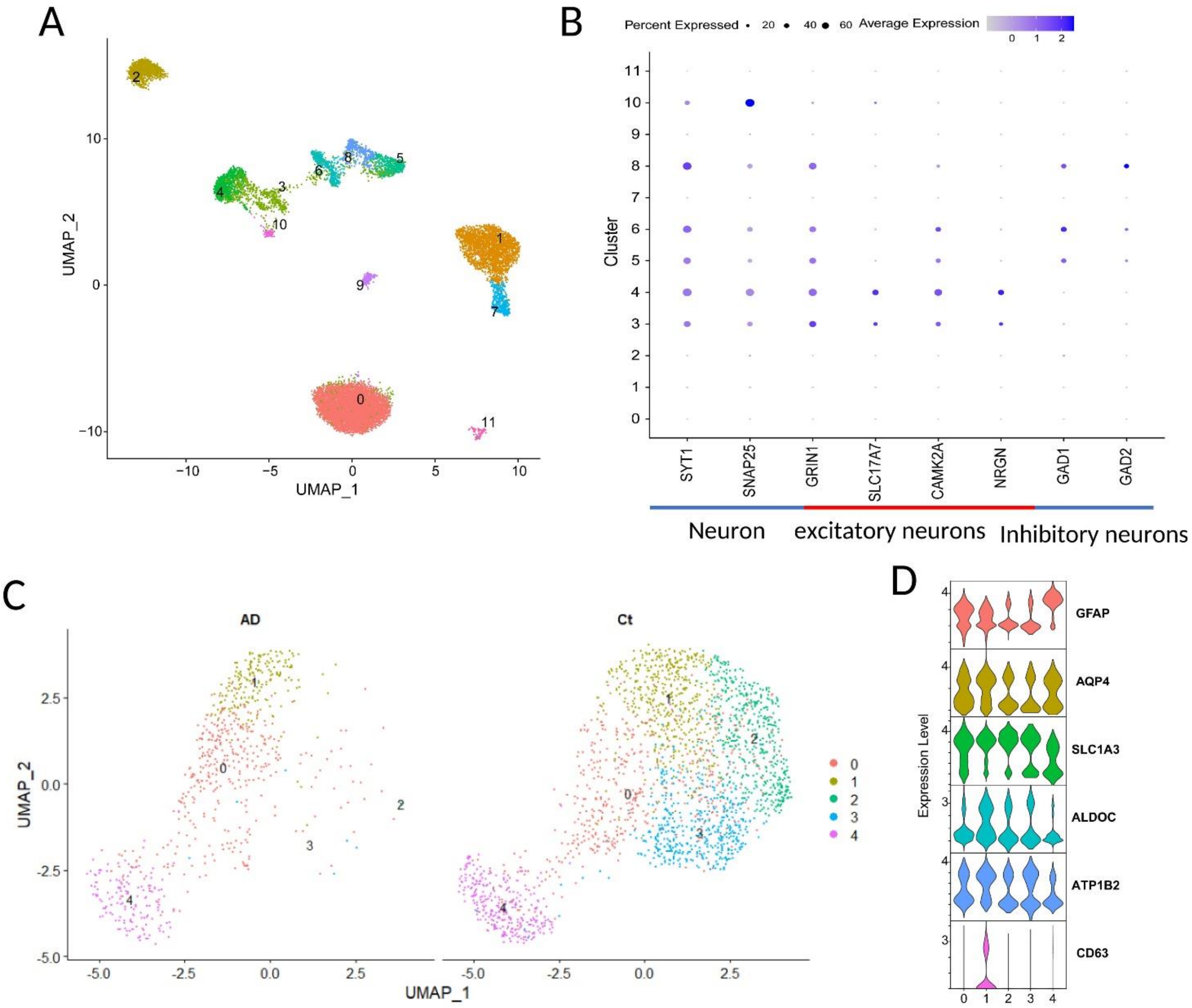

Supplementary Figure 4. cell type clusters identified from analyzing single nuclei RNA-seq datasets from GSE138852. (A) UMAP plots of all cells from dataset GSE138852. (B) The expression of neuron markers and proportion of clusters identified in control and AD samples. C, UMAP plots of astrocyte lineage cells separated by patient information. D, The expression of astrocyte specific marker in astrocyte lineage cells.

© 2024. Wei H et al. Published online at http://www.aginganddisease.org/EN/10.14336/AD.2024.0205-1

SUPPLEMENTARY DATA

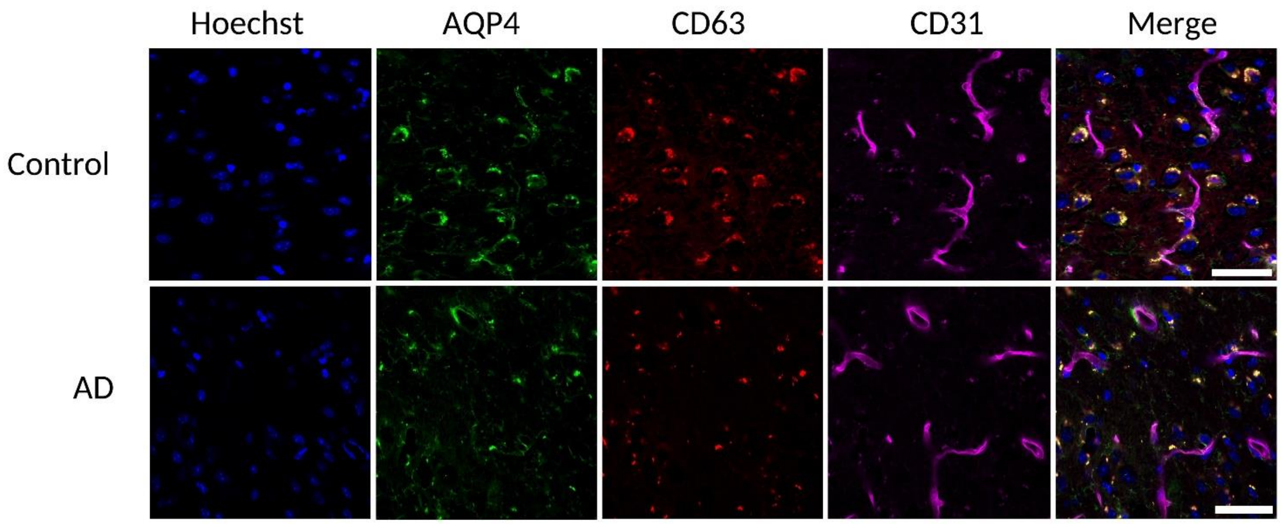

Supplementary Figure 5. The change of locations of AQP4 protein in the context of cerebral vessels in control and AD prefrontal cortex. Representative images of immunohistochemistry showing that more $AQP4^+/CD63^+$ (Green/Red) cells are located in the close vicinity of cerebral vessels marked by $CD31^+$ expression (Magenta) in 27-months old control compared to the 20-Months old $APP^{NL-F/NL-F}$ knock-in (APP^{NL-F}) mouse brain (Scale bar 50 µm).

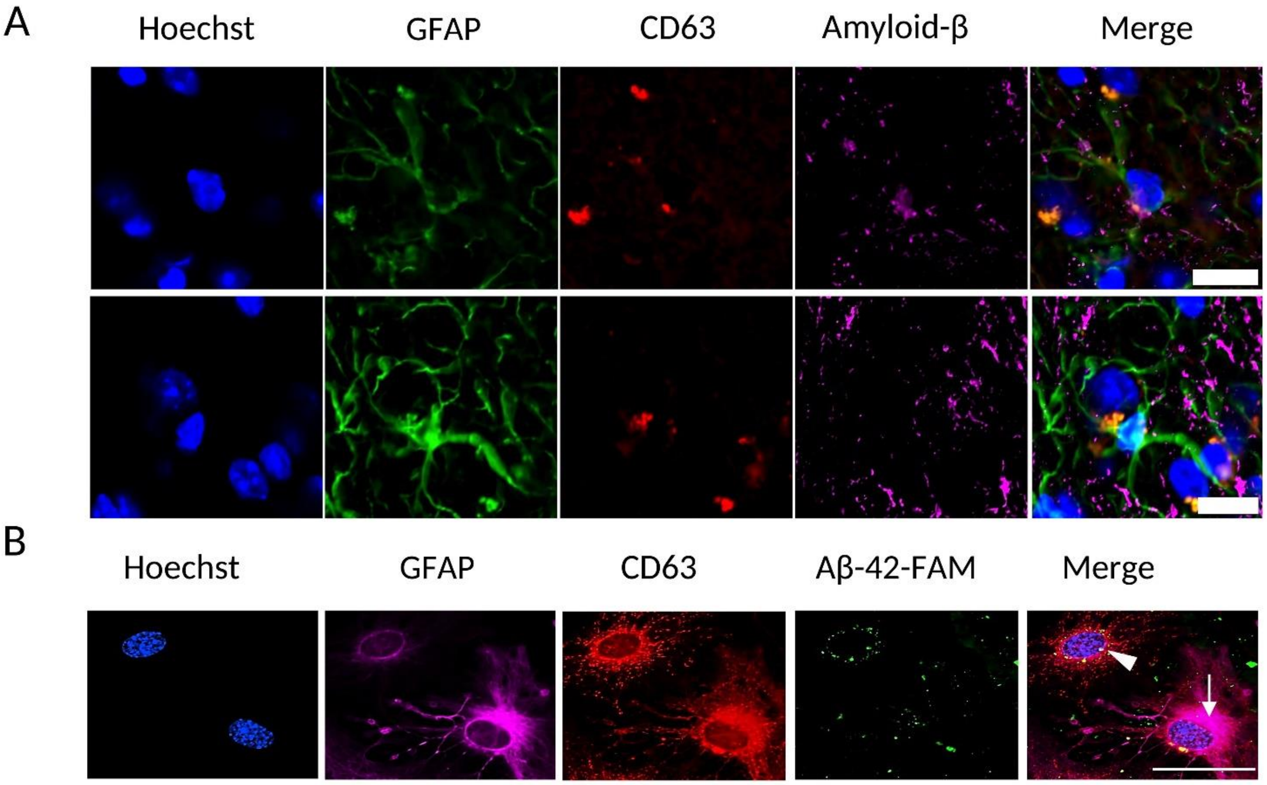

Supplementary Figure 6. Aβ plaque engulfed by $GFAP^{low}/CD63^+$ cells *in vivo* and *in vitro*. (A) Immunohistochemistry of 20-months old $APP^{NL-F/NL-F}$ knock-in (APP^{NL-F}) AD mouse brain prefrontal cortex showing colocalization of Aβ plaque with $GFAP^{low}/CD63^+$ cells (upper panel), in contrast to $GFAP^{high}/CD63^+$ cells (lower panel) (Scale bar 100 µm). (B) Immunocytochemistry of primary astrocyte culture with FAM labeled Aβ42 showing engulfment of FAM-Aβ42 by $GFAP^{low}/CD63^+$ cells compared with $GFAP^{high}/CD63^+$ cells (Scale bar 100 µm). Arrowheads indicate $GFAP^{low}/CD63^+$ astrocytes and Arrows indicate $GFAP^{high}/CD63^+$ astrocytes.

© 2024. Wei H et al. Published online at http://www.aginganddisease.org/EN/10.14336/AD.2024.0205-1
